# Characteristics and correlations of sleep disorders in patients with relapsing–remitting multiple sclerosis in China: a cross-sectional study

**DOI:** 10.3389/fneur.2025.1608802

**Published:** 2025-07-17

**Authors:** Rongrong Wang, Tengteng Zhang, Han Wang, Yao Ren, Runze Zhao, Gaopan Zhang, Guoxun Zhang, Xiongfei Zhao

**Affiliations:** ^1^Xianyang Hospital of Yan 'an University, Xianyang, China; ^2^Xijing Hospital, Air Force Medical University, Xi’an, China

**Keywords:** relapsing–remitting multiple sclerosis, sleep disorders, fatigue, anxiety, depression, cognitive impairment

## Abstract

**Background:**

Sleep disorders are a major but overlooked symptom in patients with multiple sclerosis (MS).

**Objectives:**

This article aims to investigate the characteristics of sleep disorders in patients with relapsing–remitting multiple sclerosis (RRMS) and to analyze the correlations between sleep disorders in RRMS and anxiety, depression, fatigue, and cognitive impairment.

**Methods:**

A total of 35 patients with RRMS and 35 controls were included, and both groups underwent assessments for sleep, anxiety, depression, fatigue, and cognitive function.

**Results:**

The RRMS group and the control group showed significant differences in Pittsburgh Sleep Quality Index (PSQI), Athens Insomnia Scale (AIS), Insomnia Severity Index (ISI), Hamilton Anxiety Scale (HAMA), Hamilton Depression Scale (HAMD), Modified Fatigue Impact Scale (MFIS), and Montreal Cognitive Assessment (MoCA). The group with poor sleep quality (PSQI > 5) had significantly higher scores on the AIS, ISI, HAMA, and HAMD Scale compared to the group with good sleep quality (*p* = 0.036, *p* < 0.001, *p* = 0.036, *p* = 0.054). The PSQI showed a negative correlation with disease duration; the PSQI showed a positive correlation with HAMA, HAMD, and Activities of Daily Living (ADL); AIS, ISI, and Sleep Hygiene Awareness and Practice Scale (SHAPS) all demonstrated significant positive correlations with MFIS, HAMA, and HAMD; Dysfunctional Beliefs and Attitudes about Sleep Scale (DBAS) showed a negative correlation with HAMA and HAMD.

**Conclusion:**

Sleep disorders, fatigue, anxiety, depression, and cognitive impairments are more likely to occur in patients with RRMS; there is a certain correlation between PSQI, AIS, ISI, SHAPS, and DBAS scores in the RRMS group and fatigue, anxiety, and depression.

## Introduction

Multiple Sclerosis (MS) is a chronic, inflammatory, and neurodegenerative disease of the central nervous system characterized by demyelination involving the white matter, cortex, and subcortical gray matter. The pathogenesis includes axonal loss and demyelination (1). Relapsing–remitting multiple sclerosis (RRMS) is the most common form of the disease in clinical practice, accounting for about 85% of cases. The incidence and prevalence of MS are on the rise in both developed and developing countries (2), with about 2.8 million people affected worldwide, making it the most common cause of non-traumatic disability in young individuals (3). In China, the overall incidence of MS in the population is 0.235 per 100,000 person-years, with a male-to-female patient ratio of 1:2.02 (4). In recent years, research on the correlation between sleep disorders and MS has become a focal point of interest both domestically and internationally. Sleep disorders are prevalent in Patients with MS, accounting for 60% of adult MS patients reporting sleep difficulties (5). It is noteworthy that sleep disorders can further exacerbate other consequences of MS, such as cognitive impairments, cardiovascular function, and functional capacity (5), and the growing evidence suggests that sleep disorders can negatively impact the course of MS (6). Additionally, evidence suggests that subjective sleep quality significantly predicts the physical and psychological quality of life (7). However, despite their frequent occurrence, sleep disorders are often unrecognized by medical professionals, which can significantly affect their well-being and quality of life. To date, the majority of assessment tools for sleep disorders have relied solely on the Pittsburgh Sleep Quality Index (PSQI), which lacks a comprehensive set of criteria for evaluating sleep disorders. Therefore, this study intends to employ multiple relevant scales to conduct a comprehensive assessment of sleep, hence the use of the PSQI to measure the sleep quality of patients over the past month, the Athens Insomnia Scale (AIS) to measure the quality and quantity of patients’ sleep and the daily life situation of insomnia patients in the past month, the Insomnia Severity Index (ISI) to measure the degree of insomnia in patients in the past 2 weeks, the Dysfunctional Beliefs and Attitudes about Sleep Scale (DBAS) to measure people’s identification with sleep-related thoughts and attitudes toward sleep, the Epworth Sleeping Scale (ESS) to measure patients’ sleepiness status in recent months, and the Sleep Hygiene Awareness and Practice Scale (SHAPS) to measure whether people’s daily activities are beneficial to sleep. Patient data will be organized and statistically analyzed in comparison with the control group data to identify the relevant influencing factors of sleep disorders, to intervene in the modifiable factors of sleep disorders at an early stage, and to provide guidance for the effective management of clinical symptoms of sleep disorders.

## Data and methods

### Study subjects

A total of 52 patients with MS who visited Xianyang Hospital of Yan’an University from January 1, 2023, to December 31, 2023, were selected. After excluding 17 cases that did not meet the inclusion criteria, 35 MS cases were finally included. At the same time, 35 healthy volunteers with an age difference of ±2 years, the educational level differs by ±3 years, and a gender ratio comparable to the relapsing–remitting MS group were selected as the control group. This study involved human participants, and all study procedures were conducted in accordance with the principles of the Declaration of Helsinki. All subjects gave their informed consent and the study was approved by the Ethics Committee of Xianyang Hospital of Yan’an University (Ethical Approval Number: YDXY-KY-2022-030).

1. Inclusion criteria were as follows: (a) Patients with relapsing–remitting MS who visited Xianyang Hospital of Yan’an University from January 1, 2023, to December 31, 2023; (b) All RRMS patients were diagnosed according to the revised McDonald criteria in 2017 (8); (c) Age ≥18 years old.
2. Exclusion Criteria: (a) Patients with incomplete clinical data or lost to follow-up; (b) Other types of MS; (c) Patients with impaired vision, speech, hearing, and other functional disorders affecting the assessment of the scale; (d) Patients with other diseases that may affect sleep disorders (such as migraine, tumor, Alzheimer’s disease, etc.); (e) patients currently or previously suffering from mental illness and dependent on psychotropic drugs, RRMS patients who have had a relapse in the past 4 weeks or have received glucocorticoid therapy.

### Research content and methods

A total of 35 RRMS patients and 35 control subjects were finally included. The name, gender, age, address, occupation, underlying diseases, type of disease, course of disease, category of medication used, and duration of medication use of patients and control subjects were collected. In addition, PSQI, AIS, ISI, DBAS, ESS, SHAPS, Modified Fatigue Impact Scale (21MFIS), Hamilton Anxiety Scale (HAMA), Hamilton Depression Scale (HAMD), Montreal Cognitive Assessment (MoCA), Mini-Mental State Examination (MMSE), Activities of Daily Living (ADL), etc., were collected. The same neurologist who was professionally trained in all scales strictly followed the assessment standards of each scale and conducted the scale assessment by asking questions to the subjects in a separate clinic room. All scales were administered simultaneously during the same visit to ensure consistency and comparability of the assessment results. This approach minimizes the potential for temporal variations to affect the data. The entire test takes 1.5 h to complete.

### Clinical assessment

(1) Sleep Disorder Assessment

- PSQI: The PSQI is one of the most widely used tools for assessing sleep quality (9). It has been extensively applied in the Chinese population for evaluating sleep quality and has demonstrated good reliability and validity in multiple studies. Scores range from 0 to 21, with higher scores indicating poorer sleep quality. This study designated: PSQI scores ≤5 points as no sleep disorder, 6 to 10 points as mild sleep disorder, 11 to 15 points as moderate sleep disorder, and 16 to 21 points as severe sleep disorder.
- AIS: The AIS consists of 8 items, with the first 5 items used to assess nocturnal sleep disturbances and the last 3 items focusing on the impact of insomnia on patients’ daily lives (10). The AIS assessment period is 1 month, using a 0 to 3-level scoring form. This study designated: a total score of 0 to 3 points as normal sleep; 4 to 6 points as possible insomnia; and ≥7 points as insomnia.
- ISI: The Insomnia Severity Index (ISI): This scale is a widely used questionnaire designed to assess the presence of insomnia in individuals as well as the impact of insomnia on their quality of life (11). The scale is divided into 7 items, with a total score of 28 points. This study designated: 0 to 7 points as clinically insignificant insomnia; 8 to 14 points as subclinical insomnia (mild); 15 to 21 points as clinical insomnia (moderate); and 22 to 28 points as clinical insomnia (severe).
- DBAS: The DBAS is used to assess the degree to which individuals endorse dysfunctional beliefs and attitudes about sleep. The lower the total score, the more obvious the patient’s corresponding erroneous beliefs or behaviors, the higher the risk of chronic insomnia, and the greater the need for psychological treatments such as cognitive-behavioral therapy.
- ESS: ESS was developed by Professor Murray at Epworth Hospital in Australia and is one of the internationally recognized and commonly used subjective assessment tools for sleepiness (12). The ESS includes 8 items that easily induce sleepiness, and subjects assess the degree of drowsiness through self-assessment. Each item is scored from 0 to 3 points, with a total score of 0 to 24 points. This study designated: ESS total score >6 points indicates drowsiness, >11 points indicates excessive sleepiness, and >16 points indicates dangerous sleepiness.
- SHAPS: The SHAPS consists of three parts: sleep hygiene knowledge, sleep hygiene practices, and caffeine knowledge (10). Each item of the scale is rated from 0 to 7, with a total score ranging from 0 to 105 points. The higher the score, the poorer the sleep hygiene condition.

(2) 21MFIS

The MFIS is used to evaluate the fatigue status of patients 1 month prior. Each item uses a 5-point Likert scale, scored from 0 to 4 according to the frequency of symptoms from “none” to “almost always,” with a total score ranging from 0 to 84 points. The higher the score, the more severe the patient’s fatigue. This study designated: an MFIS total score ≥38 points as being in a state of fatigue.

(3) Cognitive Function Assessment

- MMSE: The MMSE is concise, including six aspects, with a total score of 30 points, and is the preferred scale for dementia screening. This study designated: the highest score is 30 points; a score of 27–30 points is normal; and a score <27 indicates cognitive dysfunction.
- MoCA: MoCA includes execution, visual space, naming, memory, attention, language, abstraction, recall, and orientation, with a total score of 30 points. This study designated: ≥26 points as normal, and <26 points as cognitive dysfunction.
- ADL: ADL plays an important role in assessing the patient’s daily living ability. This study designated: 100 points as independent; 75–95 points as mildly independent; 50–70 points as moderately dependent; 25–45 points as severely dependent; and 0–20 points as completely dependent.

(4) Anxiety and Depression Assessment: HAMA, HAMD-24 are designed to assess and quantify the severity of anxiety and depressive symptoms in patients over the past week.

- (HAMA) Result Analysis: <7 points is normal. >7 points may have anxiety, >14 points definitely have anxiety, >21 points indicate obvious anxiety, and >29 points indicate severe anxiety.
- (HAMD-24) Result Analysis: <8 points is normal, >20 points may be light or moderate depression, and >35 points indicate severe depression.

### Statistical methods

SPSS 26.0 statistical software was used for analysis. Quantitative data conforming to the normal distribution are represented by mean ± standard deviation (x ± s), and non-conforming data are represented by median (median, M) and interquartile range (IQR). Comparisons between the two groups were made using two independent sample t-tests or Mann–Whitney U tests. Categorical data are represented by cases (%), and group comparisons are made using chi-square tests. When the quantitative data meet the normal distribution, Pearson’s correlation analysis is used; otherwise, Spearman’s correlation analysis is used; the correlation between categorical data and quantitative data uses Spearman’s correlation analysis. *p* < 0.05 indicates a statistically significant difference.

### Research process flowchart

Figure 1.

**Figure 1:**
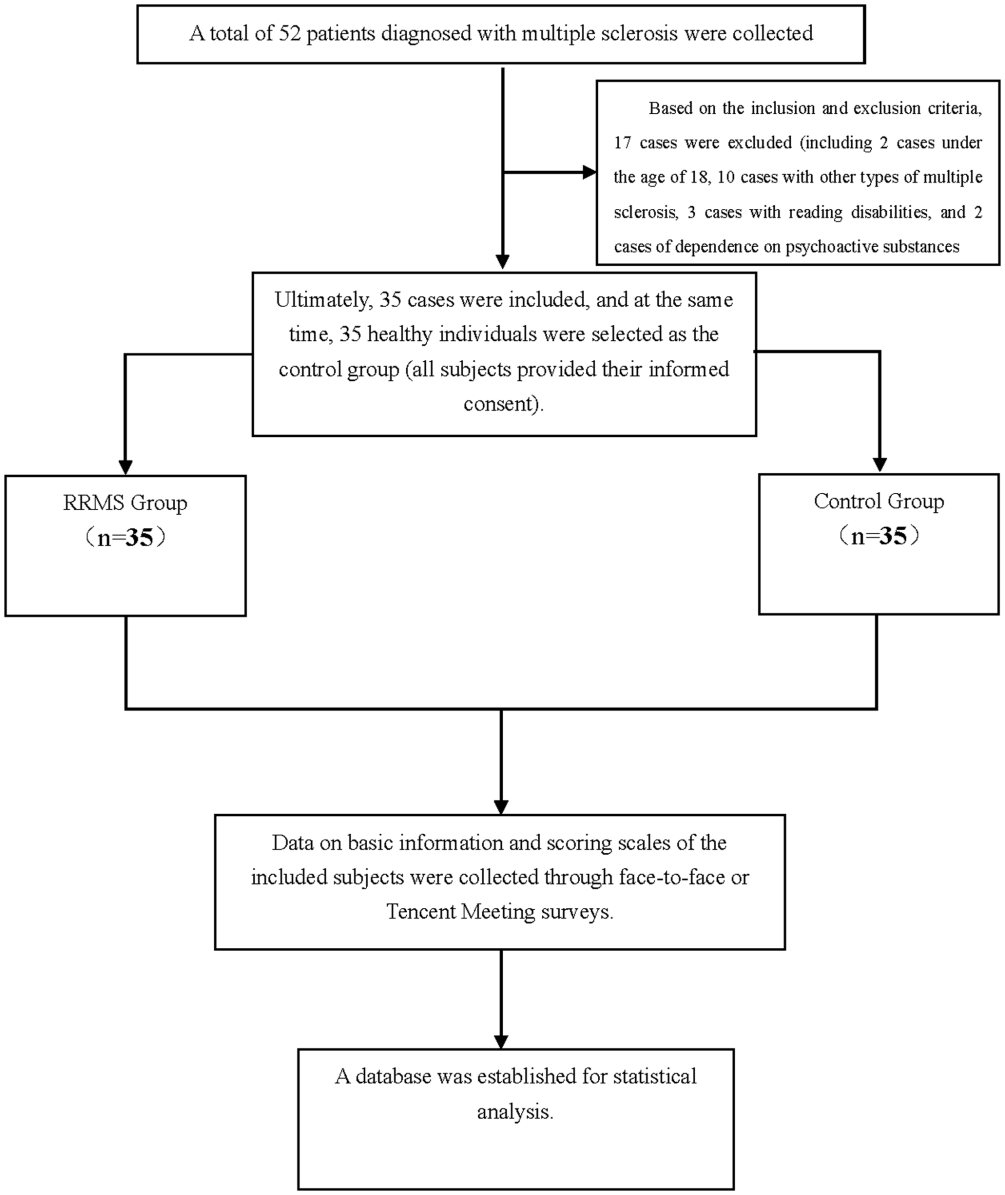
Study analysis flowchart.

## Result

### Demographic and clinical characteristics

This study included 35 cases in both the RRMS group and the control group, consisting of 9 males and 26 females, with a male to female ratio of approximately 1:3. The median age of the RRMS group was 32 (29.00, 42.00) years old (Table 1).

**Table 1:** Demographic and clinical characteristics.

Clinical data	RRMS group(*n* = 35)	Control group(*n* = 35)	*p-*value
Gender [Example (%)]			
Male	9(25.71)	9(25.71)	1.00
Female	26(74.29)	26(74.29)	
Age (years)	32(29,42)	32(28,37)	0.285
Educational level [Number (%)]			
Elementary School	1(2.86)	2(5.71)	0.818
Junior High School	2(5.71)	1(2.86)	
High School	7(20.00)	9(25.71)	
Bachelor’s Degree and Above	25(71.43)	23(65.71)	
Marital status [Example (%)]			
Married	26(74.29)	28(80.00)	0.569
Unmarried	9(25.71)	7(20.00)	

*p <* 0.05 indicates statistical significance.

### Comparison of sleep disorders between RRMS group and control group

Using the PSQI scoring scale, 13 RRMS patients (37.14%) reported mild sleep disturbances, 5 RRMS patients (14.29%) reported moderate sleep disturbances, and 1 RRMS patient (2.86%) reported severe sleep disturbances, whereas in the control group, 5 individuals (14.29%) reported mild sleep disturbances, with no moderate or severe sleep disturbances reported (Table 2). Using the AIS scoring, 13 RRMS patients (37.14%) and 1 control group subject (2.86%) reported insomnia (Table 2). Using the ISI scoring scale, 10 RRMS patients (28.57%) reported mild insomnia, and 5 RRMS group patients (14.29%) reported moderate insomnia, while the control group had no reported insomnia (Table 2). Using the ESS scoring, 8 RRMS patients (22.86%) and 3 control group subjects (8.57%) reported excessive daytime sleepiness. There were significant differences between the RRMS group and the control group in terms of PSQI, AIS, and ISI scores (*p* < 0.05; Table 2).

**Table 2:** Comparison of sleep disorders between relapsing–remitting multiple sclerosis group and control group.

Assessment scales	RRMS group(*n* = 35)	Control group(*n* = 35)	*p-*value
PSQI			
None (PSQI score < 5)	16(45.71)	30(85.71)	0.003
Mild (PSQI score 6–10)	13(37.14)	5(14.29)	
Moderate (PSQI score 11–15)	5(14.29)	0(0.00)	
Severe (PSQI score 16–21)	1(2.86)	0(0.00)	
AIS			
Normal Sleep	16(45.71)	24(68.57)	0.002
Suspected Insomnia	6(17.14)	10(28.57)	
Insomnia	13(37.14)	1(2.86)	
ISI			
None	20(57.14)	35(100.00)	<0.001
Mild	10(28.57)	0(0.00)	
Moderate	5(14.29)	0(0.00)	
Severe	0(0.00)	0(0.00)	
ESS			
No Drowsiness	18(51.43)	19(54.29)	0.212
Drowsy	9(25.71)	11(31.43)	
Excessive Drowsiness	8(22.86)	3(8.57)	
Dangerous Drowsiness	0(0.00)	2(5.71)	

*p <* 0.05 indicates statistical significance. PSQI, Pittsburgh Sleep Quality Index; AIS, Athens Insomnia Scale; ISI, Insomnia Severity Index; ESS, Epworth Sleepiness Scale.

### Comparison of anxiety, depression, fatigue, and cognition between RRMS group and control group

As shown in Table 3, the analysis revealed that 15 RRMS patients (42.86%) did not report anxiety, 2 RRMS patients (5.71%) had significant anxiety, and 2 RRMS patients (5.71%) had severe anxiety, whereas 34 control group subjects (97.14%) did not report anxiety. 14 RRMS patients (40.00%) did not report depression, 6 RRMS patients (17.14%) definitely had depression, and 3 RRMS patients (8.57%) had severe depression, while 34 control group subjects (94.29%) did not report depression. Using the MFIS cutoff score of 38, 13 RRMS patients (37.14%) and 1 control group subject (2.86%) reported significant fatigue (Table 3). Using the MoCA scoring scale, 14 RRMS patients (40.00%) and 6 control group subjects (17.14%) reported cognitive impairment. Using the MMSE scoring scale, 4 RRMS patients (11.43%) and 1 control group subject (1.43%) reported cognitive impairment (Table 3). Using the ADL scoring scale, 3 RRMS patients (8.57%) and 2 RRMS patients (5.71%) reported mild dependence and moderate dependence. There were significant differences in anxiety, depression, fatigue, and MoCA between the RRMS group and the control group (*p* < 0.05; Table 3).

**Table 3:** Comparison of anxiety, depression, fatigue and cognition between relapsing–remitting multiple sclerosis group and control group.

Assessment scales	RRMS group(*n* = 35)	Control group(*n* = 35)	*p-*value
MFIS			
No Fatigue	22(62.86)	34(97.14)	<0.001
Fatigue Present	13(37.14)	1(2.86)	
HAMA			
Normal	15(42.86)	34(97.14)	<0.001
Possible Anxiety	10(28.57)	1(2.86)	
Probable Anxiety	6(17.14)	0(0.00)	
Definite Anxiety	2(5.71)	0(0.00)	
Severe Anxiety	2(5.71)	0(0.00)	
HAMD			
Normal	14(40.00)	33(94.29)	<0.001
Possible Depression	12(34.29)	2(5.71)	
Probable Depression	6(17.14)	0(0.00)	
Severe Depression	3(8.57)	0(0.00)	
MOCA			
Cognitive Impairment Present	14(40.00)	6(17.14)	0.034
Normal	21(60.00)	29(82.86)	
MMSE			
Cognitive Impairment Present	4(11.43)	1(1.43)	0.164
Normal	31(88.57)	34(97.14)	
ADL			
Independent	30(85.71)	35(100.00)	0.068
Mild Dependence	3(8.57)	0(0.00)	
Moderate Dependence	2(5.71)	0(0.00)	
Severe Dependence	0(0.00)	0(0.00)	
Complete Dependence	0(0.00)	0(0.00)	

*p <* 0.05 indicates statistical significance. MFIS, Multidimensional Fatigue Inventory; HAMA, Hamilton Anxiety Scale; HAMD, Hamilton Depression Scale; MoCA, Montreal Cognitive Assessment; MMSE, Mini-Mental State Examination; ADL, Activities of Daily Living.

### Comparison of sleep disorders, insomnia, daytime sleepiness, fatigue, anxiety, depression, and cognition between good and poor sleep quality in the RRMS group

The total PSQI score was divided into two groups: good sleep quality (PSQI ≤ 5 points) and poor sleep quality (PSQI total score > 5 points), and demographic analysis was conducted, followed by comparisons with insomnia, daytime sleepiness, fatigue, anxiety, depression, and cognition. The analysis showed that there were no significant differences in gender and age between the good and poor sleep quality groups (*p* > 0.05). The poor sleep quality group had significantly higher scores on the AIS, Insomnia Severity Index, and measures of anxiety and depression compared to the good sleep quality group (*p* = 0.036, *p* < 0.001, *p* = 0.036, *p* = 0.054; Table 4).

**Table 4:** Comparison of sleep disorders with insomnia, daytime sleepiness, fatigue, anxiety, depression and cognition in RRMS group.

Clinical data	RRMS group (*n* = 35)	Control group(*n* = 35)	*p-*value
Age (years)	36.06 ± 7.21	34.42 ± 11.5	0.220
Gender [Example (%)]			
Male	12(75.00%)	14(73.68)	0.929
Female	4(25.00)	5(26.32)	
Assessment scale			
AIS	1(2.5,5)	3(7,11)	0.036
ISI	0(2,6)	5(9,15)	0.001
ESS	8.06 ± 3.71	6.84 ± 4.55	0.528
DBAS	102.31 ± 26.9	91.58 ± 16.39	0.253
SHAPS	22 ± 11.13	28.58 ± 12.11	0.647
21MFIS	27.56 ± 16.26	32.16 ± 16.89	0.467
Physical Component Scale	6(12,17.75)	6(18,24)	0.370
Mental Component Scale	12.69 ± 9.8	14.74 ± 7.58	0.314
Psychosocial Component Scale	1(2,3)	0(2,4)	0.960
HAMA	1.25(5.5,8.75)	4(10,19)	0.036
HAMD	2(7.5,11)	4(15,27)	0.054
MoCA	25.63 ± 2.9	25.63 ± 1.77	0.091
MMSE	26.25(28.5,29)	28(28,29)	0.716
ADL	90(100,100)	100(100,100)	0.0.010

*p <* 0.05 indicates statistical significance. PSQI, Pittsburgh Sleep Quality Index; AIS, Athens Insomnia Scale; ISI, Insomnia Severity Index; ESS, Epworth Sleepiness Scale; DBAS, Dysfunctional Beliefs and Attitudes about Sleep Scale; SHAPS, Sleep Hygiene Awareness, Perception, and Practice Scale; MFIS, Multidimensional Fatigue Inventory; HAMA, Hamilton Anxiety Scale; HAMD, Hamilton Depression Scale; MoCA, Montreal Cognitive Assessment; MMSE, Mini-Mental State Examination; ADL, Activities of Daily Living.

### Correlation analysis of sleep disorders with age and disease duration in the RRMS group

The analysis demonstrates a negative correlation between PSQI scores and disease duration (*r* = −0.344, *p* = 0.043; Table 5).

**Table 5:** Correlation analysis of sleep disorder with age and course of disease in relapsing–remitting multiple sclerosis group.

Clinical data		Gender	Gender	Education level	Marital status	Disease duration	Medication category	Duration of medication use
PSQI	r	0.140	0.042	−0.113	0.221	−0.344	−0.057	−0.196
	p	0.423	0.812	0.517	0.202	0.043	0.747	0.259
AIS	r	0.328	−0.057	−0.172	0.156	−0.316	0.060	−0.167
	p	0.054	0.745	0.324	0.371	0.064	0.733	0.339
ISI	r	0.185	−0.170	−0.040	0.175	−0.314	0.012	−0.165
	p	0.287	0.329	0.822	0.314	0.067	0.944	0.343
ESS	r	0.277	0.302	−0.291	0.023	−0.008	0.090	−0.166
	p	0.107	0.078	0.090	0.897	0.964	0.606	0.341
DBAS	r	−0.262	−0.043	−0.133	−0.052	0.070	−0.042	0.126
	p	0.128	0.804	0.445	0.767	0.689	0.809	0.472
SHAPS	r	0.285	−0.089	−0.062	0.149	−0.056	0.244	0.020
	p	0.097	0.612	0.725	0.393	0.749	0.158	0.911

*p <* 0.05 indicates statistical significance. PSQI, Pittsburgh Sleep Quality Index; AIS, Athens Insomnia Scale; ISI, Insomnia Severity Index; ESS, Epworth Sleepiness Scale; DBAS, Dysfunctional Beliefs and Attitudes about Sleep Scale; SHAPS, Sleep Hygiene Awareness, Perception, and Practice Scale.

### Correlation analysis of sleep disorders with fatigue, anxiety, depression, etc., in the RRMS group

The analysis showed that the PSQI score was positively correlated with HAMA (*r* = 0.453, *p* = 0.006) and HAMD (*r* = 0.479, *p* = 0.004), ADL (*r* = 0.406, *p* = 0.016); AIS was significantly positively correlated with MFIS (*r* = 0.640, *p* = 0.000), HAMA (*r* = 0.681, *p* = 0.000), HAMD (*r* = 0.732, *p* = 0.000); ISI was significantly positively correlated with MFIS (*r* = 0.436, *p* = 0.009), HAMA (*r* = 0.600, *p* = 0.000), HAMD (*r* = 0.567, *p* = 0.000); DBAS was negatively correlated with HAMA (*r* = −0.354, *p* = 0.037), HAMD (*r* = −0.463, *p* = 0.005); SHAPS was significantly positively correlated with MFIS (*r* = 0.554, *p* = 0.001), HAMA (*r* = 0.620, *p* = 0.000), HAMD (*r* = 0.579, *p* = 0.000; Table 6).

**Table 6:** Correlation analysis of sleep disorders with fatigue, anxiety and depression in relapsing–remitting multiple sclerosis group.

Clinical data	PSQI		AIS		ISI	
	r	p	r	p	r	p
21MFIS	0.217	0.210	0.640	0.000	0.436	0.009
Physical Component Scale	0.151	0.387	0.501	0.002	0.383	0.023
Mental Component Scale	0.237	0.170	0.690	0.000	0.447	0.007
Psychosocial Component Scale	0.077	0.661	0.300	0.080	0.193	0.267
HAMA	0.453	0.006	0.681	0.000	0.600	0.000
HAMD	0.479	0.004	0.732	0.000	0.567	0.000
MoCA	−0.172	0.322	−0.208	0.230	−0.120	0.492
MMSE	−0.057	0.746	−0.305	0.075	−0.131	0.453
ADL	0.406	0.016	0.106	0.546	0.314	0.067
	ESS		DBAS		SHAPS	
21MFIS	0.310	0.070	−0.268	0.120	0.554	0.001
Physical Component Scale	0.264	0.125	−0.297	0.084	0.432	0.010
Mental Component Scale	0.234	0.175	−0.191	0.272	0.528	0.001
Psychosocial Component Scale	0.405	0.016	−0.125	0.473	0.416	0.013
HAMA	0.037	0.833	−0.354	0.037	0.620	0.000
HAMD	0.025	0.888	−0.463	0.005	0.579	0.000
MoCA	0.119	0.496	−0.194	0.265	−0.154	0.377
MMSE	0.134	0.444	0.083	0.635	−0.228	0.187
ADL	−0.208	0.231	−0.111	0.526	−0.186	0.286

*p <* 0.05 indicates statistical significance. PSQI, Pittsburgh Sleep Quality Index; AIS, Athens Insomnia Scale; ISI, Insomnia Severity Index; ESS, Epworth Sleepiness Scale; DBAS, Dysfunctional Beliefs and Attitudes about Sleep Scale; SHAPS, Sleep Hygiene Awareness, Perception, and Practice Scale; MFIS, Multidimensional Fatigue Inventory; HAMA, Hamilton Anxiety Scale; HAMD, Hamilton Depression Scale; MoCA, Montreal Cognitive Assessment; MMSE, Mini-Mental State Examination; ADL, Activities of Daily Living.

## Discussion

In this study, we analyzed the relevant characteristics of sleep disorders in RRMS patients, identified the influencing factors of sleep disorders, and looked for factors that affect sleep to help manage sleep problems better. In terms of gender distribution, the ratio of male to female RRMS patients included in our study was approximately 1:3, but there was no significant difference in sleep quality between men and women. This finding is contrary to the results of the study by B ø e Lunde et al. (13), and similar to the results of Turkowitch D (10) et al. (14), indicating that there is no significant correlation between gender and sleep quality in patients with RRMS. Regarding the age of patients, Flügel D et al.’s study showed that sleep disorders in Patients with MS are somewhat related to age, with the incidence of sleep disorders increasing and severity positively correlated with age (15), Additionally, as age increases, the production of hormones decreases, which is negatively correlated with sleep quality, suggesting that melatonin deficiency at least partially contributes to sleep disorders (16). However, our study showed no significant correlation between sleep disorders and age, which may be related to our small sample size, and further research with a larger sample size is needed. A review of the literature for this study indicates that poor sleep is more common in women and patients with a longer disease course (17, 18). Our research indicates that poor sleep quality is inversely correlated with the duration of the disease, which aligns with the findings of Zhang GX et al., suggesting that patients with prolonged disease courses are more likely to accept the symptoms of the illness, including sleep disturbances (19). In this study, it can be interpreted that MS is more prevalent in young people, and as the disease progresses, patients gradually get used to life, thus reducing severe sleep disorders.

The prevalence of poor sleep quality in Patients with MS is relatively high, with previous studies showing a prevalence of 47 to 62% (20), In Kotterba S et al.’s prospective study of 73 RRMS or CIS patients, the proportion of poor sleep quality was about 50%. Our data showed that the prevalence of sleep disorders in RRMS patients reaches 54.29% among MS patients, which is about 2.8 times that of the control group, and patients with moderate to severe sleep quality are significantly higher than the control group, which is consistent with the published data. Therefore, sleep disorders are a common comorbidity in patients with MS, with approximately half of the patients being troubled by them, a proportion significantly higher than in the population not affected by MS. This difference may be because of small study samples and different definitions and assessment methods for sleep disorders.

Observations indicated that the prevalence of insomnia is high among individuals diagnosed with MS, with multiple studies reporting that the prevalence of insomnia in Patients with MS was approximately 40% (21), often manifesting as early morning awakenings. Compared to the general population, there is less literature on the prevalence of insomnia in Patients with MS. However, observational studies indicate that the rate of insomnia in Patients with MS is higher than in the general population. Our study reports that the prevalence of insomnia in the RRMS group reaches 37.14% among MS patients, among 35 RRMS patients, 28.57% had mild insomnia, and 14.29% had moderate insomnia, with a significantly higher prevalence and severity index compared to the control group, consistent with the aforementioned research results. However, there are currently no specific guidelines for the treatment of insomnia in Patients with MS. A treatment study showed that treating depressive states in Patients with MS can improve insomnia, but many of the drugs involved have tolerance and addiction, which may limit their long-term use (22). It has been observed that in patients diagnosed with MS, early identification and management of insomnia is a frequently overlooked factor of sleep disorders.

The results of this study show that there is no significant correlation between sleep quality (PSQI) and fatigue (MFIS) (*p* > 0.05), while insomnia (AIS/ISI) is significantly correlated with fatigue (*p* < 0.05). This discrepancy may reflect the limitations of PSQI in capturing sleep disorders related to fatigue, especially in patients with relapsing–remitting multiple sclerosis (RRMS). This is consistent with the findings of Morin CM (23), that is, PSQI is used mainly to assess overall sleep quality and is less sensitive to certain specific sleep problems (such as insomnia). In contrast, AIS and ISI focus more on insomnia symptoms, which may have a more direct link with fatigue.

This study shows that the prevalence of excessive daytime sleepiness in RRMS patients is not significantly different from the control group. This is consistent with the research results of Bøe Lunde HM and Schutte-Rodin S et al. (24, 25), Some studies found no excessive daytime sleepiness in RRMS. Some see no sleepiness difference between fatigued and non-fatigued MS patients, while others find sleepiness is closely linked to fatigue (26). Our study found that daytime sleepiness is not significantly correlated with fatigue, anxiety, depression, or cognition. Moreover, poor sleep quality in RRMS patients is not correlated with excessive daytime sleepiness, despite an increased frequency of fatigue and sleep complaints in patients with poor sleep quality.

The results of this study showed that DBAS has a significantly negative correlation with anxiety and depression, consistent with Kuhn T’s research results (27), which also confirmed that DBAS is highly correlated with depressive symptoms, insomnia severity, and anxiety symptoms, and that sleep beliefs and attitudes are negative, unrealistic worries, biases, or misconceptions related to sleep, which can perpetuate or exacerbate insomnia. The more severe the symptoms of anxiety and depression, the more obvious the irrational cognition about sleep. In our study, one explanation for how changes in sleep beliefs and attitudes affect psychological pain and fatigue can be understood through the influence of thoughts on physiological sensations, emotions, and behaviors. This can also remind neurologists to identify and guide patients to develop good sleep habits in a timely manner, such as trying to meditate or practice deep breathing before going to bed to relax, helping patients adjust their mindset, find a suitable way to relax, and seek professional guidance and treatment when necessary.

The results of this study indicated that there was a statistically significant difference in sleep hygiene habits between the RRMS group and the control group, meaning that RRMS patients have poorer sleep hygiene and need to improve their attitudes and behaviors toward sleep to change their overall sleep situation. However, there was no significant difference in sleep quality and sleep hygiene habits. This was consistent with the research results of Alshahrani M and Al-Kandari S, which did not find a significant association between sleep quality and sleep hygiene awareness (28, 29), but contraried to the research results of Suen LK et al (30). This difference may be attributed to the heterogeneity of participants, study design, and assessment tools. Moreover, the study shows that sleep hygiene habits are significantly positively correlated with fatigue, anxiety, and depression. That is, patients with more anxiety, depression, and fatigue exhibit poorer sleep hygiene conditions. Therefore, we should advocate for neurologists to pay attention to patients’ sleep hygiene habits and provide some sleep health advice.

Our study shows that about 37.14% of RRMS patients report fatigue symptoms. The research conducted by Eizaguirre et al. (31) shows that the incidence of fatigue in Patients with MS is 51.6%, which is relatively lower in our study. This may be due to the relatively shorter disease course of patients in this study, and also considering the inconsistency of scale assessment methods used in various studies, results may vary. However, compared with the control group, RRMS group patients are more prone to fatigue. A large number of epidemiological studies have shown that subjective sleep disorders in MS and the general population are closely related to fatigue symptoms (14), But in our study, the PSQI sleep quality is not correlated with fatigue (*p* ≤ 0.05), while the AIS insomnia scale and ISI are both significantly positively correlated with fatigue (*p* ≤ 0.001). This result may be related to subjective factors of the patient and the small amount of data included, and also suggests that in subsequent studies of sleep disorders in MS patients, more scales need to be included for comprehensive assessment. In addition, sleep disorders can exacerbate the fatigue of MS patients, and sleep disorders may lead to the acute exacerbation of MS. A recent randomized controlled treatment study has shown that addressing sleep disorders in MS can improve patients’ fatigue symptoms (32).

It is reported that the lifetime morbidity rate of anxiety in the MS population is 35% (33), The results of this study show that about 28.56% of RRMS patients have anxiety symptoms, which is slightly lower than previous studies, possibly due to the inconsistency of anxiety scales used in various studies, leading to differences in results. The results of this study show that the prevalence of anxiety in our RRMS group patients is significantly higher than that in the control group, indicating that compared with the healthy population, RRMS patients are more prone to anxiety symptoms. The total score of PSQI is significantly positively correlated with the Hamilton Anxiety Scale (HAMA), and both the AIS and the ISI are significantly positively correlated with HAMA, indicating that the presence of sleep disorders in RRMS patients is closely related to the level of anxiety. These findings are consistent with data provided by other researchers, who believe that anxiety and stress may be important factors in the occurrence or progression of SD due to the dysfunction of brain mediators (34). Other authors link MS with anxiety because anxiety can exist independently of disease type, disease course, disability level, or age, and it worsens cognitive function and sleep quality (35).

A multicenter cross-sectional study conducted in Italy (36) found a prevalence of depression of 33.9%. Further research shows that the prevalence of depression in patients with RRMS is 26.6%. Our study indicates that about 25.71% of RRMS patients have depressive symptoms, which is consistent with the aforementioned research results. Moreover, the prevalence of depression in the RRMS group is significantly higher than that in the control group, indicating that compared with the general population, patients with MS are more prone to mood disorders. Furthermore, PSQI, AIS, and ISI are all significantly positively correlated with depression. We revealed a positive correlation between depression and insomnia, which is consistent with the data of Bahman D. S. et al., emphasizing the significant impact of depression on sleep quality and vice versa. The presence of insomnia in depressive symptoms can further worsen (37). That is, the more severe the degree of depression, the poorer the sleep quality and the more severe the insomnia. This is similar to previous studies (24), suggesting a close link between sleep disorders and depression. Insufficient sleep can predict depression, and depressive mood may be a predictive factor for sleep interruption. Therefore, RRMS patients with poor sleep quality should be screened for depression and treated accordingly. However, the pathogenesis of depression associated with RRMS is not yet clear and may be related to genetics, brain structural changes, immune inflammation, and psychosocial factors (38).

Clinical surveys indicate that 40 to 70% of MS patients have cognitive dysfunction, which severely affects the daily functioning of MS patients (39). MS patients who report symptoms of sleep disorders may also exhibit signs of cognitive impairment (22). In our study, there were no significant differences between RRMS patients and the control group in MMSE and ADL scores, while the incidence of cognitive impairment in the RRMS group as measured by MoCA was twice that of the control group, indicating that compared to the healthy population, patients with relapsing–remitting MS are more likely to experience cognitive dysfunction. In the present study, no significant differences were observed in MMSE scores between the RRMS group and the control group, which stands in stark contrast to the findings from the MoCA. Cognitive impairments in MS patients are typically mild to moderate and are most pronounced in domains such as executive function, attention, and working memory (40). These cognitive deficits may not be adequately captured by the MMSE, whereas the MoCA has demonstrated superior sensitivity and specificity in detecting mild cognitive impairments (41). This discrepancy likely underscores the limitations of the MMSE in detecting cognitive impairments associated with MS. By categorizing the PSQI into good sleep quality (PSQI ≤ 5 points) and poor sleep quality (PSQI > 5 points), the SPSS results showed no significant difference in cognitive impairment between worsening sleep quality and other studies that report the impact of sleep quality on cognitive function (42). At the same time, all RRMS patients with severe cognitive impairment due to sleep disorders performed poorly in all cognitive domains, with memory and language being the lowest. A significant association between sleep disorders and cognitive dysfunction has been found in some studies (43). In the systematic review by Hughes, A. J., et al., memory and executive functions were found to be the most affected in patients with sleep disorders (43). Previous studies have identified sleep disorders as a predictor of future cognitive decline in MS (44) and the results of systematic reviews emphasize the necessity of incorporating sleep assessment into routine MS care. Interventions targeting sleep disorders may offer hope for improving cognitive dysfunction in MS.

Due to the increased prevalence of sleep disorders in Patients with MS, the results of this study provide evidence to support regular screening and monitoring of fatigue and sleep disorders in this patient population. Moreover, treatment of sleep disorders may have beneficial effects beyond improving sleep, such as reducing anxiety, depression, and physical fatigue, which in turn may lead to improved cognitive function. Poor sleep quality is a serious issue in Patients with MS and deserves more attention. Sleep disorders may occur independently of demographic factors such as gender and clinical-demographic factors such as high psychological burden (45). In addition, sleep insufficiency may also be influenced by emotional factors and medications used to treat RRMS. However, due to the high prevalence and potential impact of sleep insufficiency in MS, more research on MS sleep and the development of successful interventions are needed.

Of course, our study has certain limitations. First, due to the cross-sectional design of this study, we cannot obtain information on long-term changes to investigate the causal pathways that may be associated with sleep disorders in RRMS patients. Second, the cohort in this study is composed only of RRMS patients. Relapsing–remitting is the most common form of MS. Therefore, our results cannot be generalized to the prevalence of SD in progressive MS. Lastly, the number of subjects included in this study is limited, and some symptoms can be explained by demographic characteristics, when selecting the study subjects, although we endeavored to strictly adhere to the inclusion and exclusion criteria, potential biases such as selection bias and demographic homogeneity may have been introduced due to the relatively small sample size. However, the main advantage of our study is the strict inclusion criteria, and the use of multiple sleep scales to assess sleep disorders, as well as the relationship with anxiety, depression, fatigue, and cognition. All questionnaires were applied by a neurologist during a personal interview with the patient to avoid false-positive diagnoses. Based on the above shortcomings, future studies will continue to expand the sample size, continue to trace long-term prognosis, and include more confounding factors, especially in the aspects of restless leg syndrome and OSA. Enhancing the understanding of modifiable risk factors for sleep insufficiency in RRMS may be of great significance for early treatment and preventive intervention.

## Conclusion

Sleep disorders are more common in patients with RRMS than in the healthy population. Patients with poor sleep quality are more likely to experience anxiety, depression, and insomnia. The disease duration is negatively correlated with sleep quality. Patients in the RRMS group are more prone to fatigue, anxiety, depression, and cognitive impairment, which have varying degrees of impact on their quality of life. In the RRMS group, PSQI, AIS, ISI, and SHAPS scores are positively correlated with fatigue, anxiety, and depression, while DBAS scores are negatively correlated with anxiety and depression.

## Data Availability

The original contributions presented in the study are included in the article/supplementary material, further inquiries can be directed to the corresponding author.
