# Correction: Characteristics and correlations of sleep disorders in patients with relapsing–remitting multiple sclerosis in China: a cross-sectional study

**DOI:** 10.3389/fneur.2025.1669440

**Published:** 2025-08-14

**Authors:** Rongrong Wang, Tengteng Zhang, Han Wang, Yao Ren, Runze Zhao, Gaopan Zhang, Guoxun Zhang, Xiongfei Zhao

**Affiliations:** ^1^Xianyang Hospital of Yan'an University, Xianyang, China; ^2^Xijing Hospital, Air Force Medical University, Xi'an, China

**Keywords:** relapsing–remitting multiple sclerosis, sleep disorders, fatigue, anxiety, depression, cognitive impairment

Author Xiongfei Zhao Was erroneously omitted as the corresponding author. The correct corresponding author list is:

Guoxun Zhang, guoxun.zhang@hotmail.com

Xiongfei Zhao, zhaoxiongfei1973@sina.com

The original version of this article has been updated.

